# Elp3 and RlmN: A tale of two mitochondrial tail-anchored radical SAM enzymes in *Toxoplasma gondii*

**DOI:** 10.1371/journal.pone.0189688

**Published:** 2018-01-02

**Authors:** Leah R. Padgett, Jenna M. Lentini, Michael J. Holmes, Krista L. Stilger, Dragony Fu, William J. Sullivan

**Affiliations:** 1 Department of Pharmacology & Toxicology, Indiana University School of Medicine, Indianapolis, Indiana, United States of America; 2 Department of Biology, University of Rochester, Rochester, New York, United States of America; 3 Department of Microbiology and Immunology, Indiana University School of Medicine, Indianapolis, Indiana, United States of America; Institut national de la santé et de la recherche médicale - Institut Cochin, FRANCE

## Abstract

Radical *S*-adenosylmethionine (rSAM) enzymes use a 5’-deoxyadensyl 5’-radical to methylate a wide array of diverse substrates including proteins, lipids and nucleic acids. One such enzyme, Elongator protein-3 (TgElp3), is an essential protein in *Toxoplasma gondii*, a protozoan parasite that can cause life-threatening opportunistic disease. Unlike Elp3 homologues which are present in all domains of life, TgElp3 localizes to the outer mitochondrial membrane (OMM) via a tail-anchored trafficking mechanism in *Toxoplasma*. Intriguingly, we identified a second tail-anchored rSAM domain containing protein (TgRlmN) that also localizes to the OMM. The transmembrane domain (TMD) on *Toxoplasma* Elp3 and RlmN homologues is required for OMM localization and has not been seen beyond the chromalveolates. Both TgElp3 and TgRlmN contain the canonical rSAM amino acid sequence motif (CxxxCxxC) necessary to form the 4Fe-4S cluster required for tRNA modifications. In *E*. *coli*, RlmN is responsible for the 2-methlyadenosine (m^2^A) synthesis at purine 37 in tRNA while in *S*. *cerevisiae*, Elp3 is necessary for the formation of 5-methoxycarbonylmethyl-2-thiouridine (mcm^5^s^2^U) at the wobble tRNA position. To investigate why these two rSAM enzymes localize to the mitochondrion in *Toxoplasma*, and whether or not TgRlmN and TgElp3 possess tRNA methyltransferase activity, a series of mutational and biochemical studies were performed. Overexpression of either TgElp3 or TgRlmN resulted in a significant parasite replication defect, but overexpression was tolerated if either the TMD or rSAM domain was mutated. Furthermore, we show the first evidence that *Toxoplasma* tRNA^Glu^ contains the mcm^5^s^2^U modification, which is the putative downstream product generated by TgElp3 activity.

## Introduction

Radical *S-*adenosylmethionine (rSAM) enzymes utilize a [4Fe-4S] cluster and SAM to generate a 5’-deoxyadenosyl radical intermediate required for methylation reactions. These enzymes are defined by the presence of a conserved cysteine motif that coordinates the formation of an [4Fe-4S] cluster. Although these motifs vary in the number of cysteines and amino acid length, the most common motif is CxxxCxxC. Over the last decade, studies have shown that rSAM enzymes play a pivotal role in the modification of RNA [1]. RNA post-transcriptional modifications affect RNA structure and stability, which impacts translation and ultimately affects numerous downstream biological processes. The study of rSAM enzymes is required to better understand the biological implications of RNA modifications.

Virtually nothing is known about the function of rSAM domain proteins in the obligate intracellular protozoan parasite *Toxoplasma gondii*. A member of the phylum Apicomplexa and Chromalveolata supergroup, *Toxoplasma* is a promiscuous pathogen that causes significant disease in humans and livestock. In humans, toxoplasmosis is typically an opportunistic disease of immune compromised patients. The definitive host for *Toxoplasma* includes feline species, which spread the parasite through shedding of infectious oocysts that are stable in the environment for a year or more. In addition, *Toxoplasma* can form infectious cysts in the tissues of vertebrate animals, facilitating its spread to new hosts through predation. If infected for the first time while pregnant, *Toxoplasma* can transmit across the placenta from mother to child. A better understanding of parasite biology will be helpful in the design of future drug targets directed against this parasite.

We previously found that the Elongator protein-3 homologue present in *Toxoplasma gondii* (TgElp3) is essential for parasite viability [2]. TgElp3 possesses two conserved domains, a rSAM domain and lysine acetyltransferase domain, along with a unique C-terminal transmembrane domain (TMD) that is only present in the phylum Apicomplexa and select chromalveolates [2]. We further determined that TgElp3 is a tail-anchored protein present at the parasite’s outer mitochondrial membrane (OMM) [2]. In yeast, Elp3 was originally characterized as the histone acetyltransferase subunit of the RNA polymerase II Elongator complex, an important regulator of transcriptional elongation [3,4]. More recent studies have determined that Elp3 possesses a second enzymatic function as a tRNA modification enzyme that synthesizes the 5-methoxycarbonylmethyl (mcm^5^) and 5-carbamoylmethyl (ncm^5^) groups present on uridines at the wobble position in tRNA [5], an activity that relies on the protein’s rSAM domain [6]. Cells without Elp3 lack the mcm^5^U modification, resulting in inefficient translation of mRNAs enriched with -AA ending codons [5,7]. Given its location at the OMM, we hypothesized that TgElp3 is more likely to play a role in RNA modification than transcriptional elongation. However, the presence of the mcm^5^U tRNA modification has yet to be confirmed in *Toxoplasma*.

Intriguingly, our recent analysis of the entire tail-anchored protein family in *Toxoplasma* uncovered a second rSAM domain containing protein (rRNA large subunit methyltransferase gene N, TgRlmN) that also localizes to the OMM like TgElp3 [8,9]. RlmN homologues have previously been identified in bacteria and plants, but not in higher eukaryotes [6,10]. In *E*. *coli*, the RlmN methyltransferase is responsible for the methyl-2 (m^2^) modification at rRNA position A2503 in 23S rRNA and the conserved purine at position 37 in tRNA [8,11]. Purine 37 modifications are thought to stabilize the first base pair of the codon-anticodon interaction while the m^2^A 2503 rRNA modification may alter the translation arrest mechanism in response to specific peptide sequences [11]. With the loss of RlmN in *E*. *coli*, translation accuracy was disrupted due to the read through of UAG stop codons [11]. Although studies in *E*. *coli* suggest that the m^2^A nucleoside plays a pivotal role in translational accuracy, the function of RlmN in *Toxoplasma* has not been investigated.

In this study, we examined the functional significance of these two mitochondrial tail-anchored rSAM domain containing proteins (TgElp3 and TgRlmN) in *Toxoplasma*. As these proteins share similar protein characteristics and previous attempts to disrupt TgElp3 have failed [2], we used an overexpression strategy to assess their importance in parasite biology. Overexpression of TgElp3 or TgRlmN results in a significant parasite replication defect unless the rSAM domain is mutated or the TMD is removed. Recent studies regarding Elp3 and RlmN in various organisms have determined that these enzymes modify tRNAs, which regulates translation and subsequently affects protein homeostasis [11–13]. Polyribosomal profiling revealed that neither TgElp3 nor TgRlmN overexpression has a significant impact on global protein production in *Toxoplasma*. As an independent confirmation, we report the first use of puromycin incorporation to monitor global protein synthesis in *Toxoplasma*, which also found that overexpression of TgElp3 or TgRlmN did not alter translation. The course of these studies did, however, provide the first evidence that the mcm^5^s^2^U tRNA modification, which is generated by Elp3 in other species, is present in *Toxoplasma*.

## Results

### Generation of wildtype and mutant TgElp3 parasite lines

**Fig 1:**
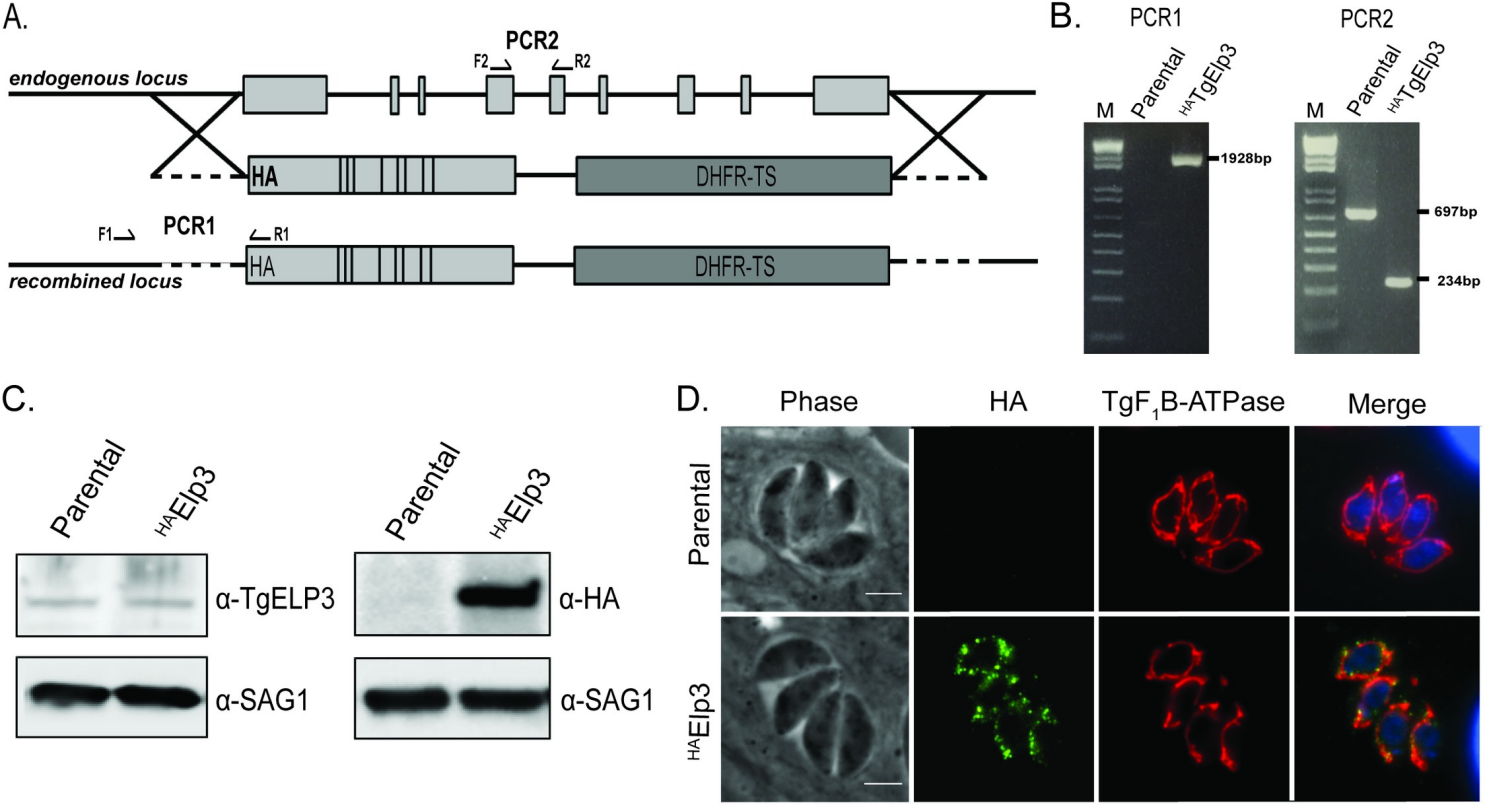
Endogenous replacement of TgElp3. (A) Diagram of the construct used to replace the endogenous TgElp3 locus by double homologous recombination. Arrows indicate location of PCR primers. For PCR1, the forward primer is located upstream of the recombination site and the reverse primer is located within the HA-tag. The PCR2 primers are intron spanning. (B) Correct integration of ^HA^TgElp3 at the endogenous locus is confirmed by genomic PCR analysis. (C) For each sample, 100 μg of protein was used for Western blot analysis of parental RHΔ*hx*Δ*ku80* and ^HA^TgElp3 parasites. The blot was probed with anti-TgELP3, anti-HA and anti-SAG1 (as a loading control) antibodies. (D) IFAs stained for anti-HA (green) and the mitochondrial maker anti-TgF_1_B ATPase (red). Images merged with the DNA stain DAPI (blue). Scale bar = 3μm.

As previous attempts to knockout TgElp3 have failed and localization of TgElp3 to the mitochondrion is essential for parasite viability, we sought to characterize the molecular function of TgElp3 [2,9]. Since TgElp3 possesses two highly conserved enzymatic domains, a radical *S*-adenosylmethionine (rSAM) and a lysine acetyltransferase (KAT) domain [2], we decided to take a targeted mutational approach to determine which domain(s) is required for TgElp3 function. As a control, we replaced the endogenous locus with hemagglutinin (HA)-tagged wild-type cDNA (^HA^TgElp3) in RHΔ*hx*Δ*ku80* parasites; recombination frequency was ~50%. Correct integration was confirmed by PCR and protein expression was assessed by Western blot using anti-TgELP3 [14] and anti-HA antibodies (Fig 1). Immunofluorescent assays (IFA) confirmed ^HA^TgElp3 localization to the parasite mitochondrion using the established mitochondrial marker F_1_B ATPase (Fig 1) [15,16].

To determine if parasites could survive when either the rSAM or KAT domains were mutated, we generated two more ^HA^TgElp3 allelic replacement constructs: rSAM mutant (C284A) and KAT mutant (Y715A/Y716A); in other species, these rSAM and KAT mutations are critical for Elp3 function [6,17–19]. Despite multiple attempts, we were unable to generate rSAM or KAT mutants at the endogenous TgElp3 locus. Since the endogenous replacement of TgElp3 with wild-type recombinant ^HA^TgElp3 was obtained, we conclude that both enzymatic domains are likely required for TgElp3 function and parasite viability.

### Overexpression of TgElp3 at the mitochondrion causes a significant replication defect

**Fig 2:**
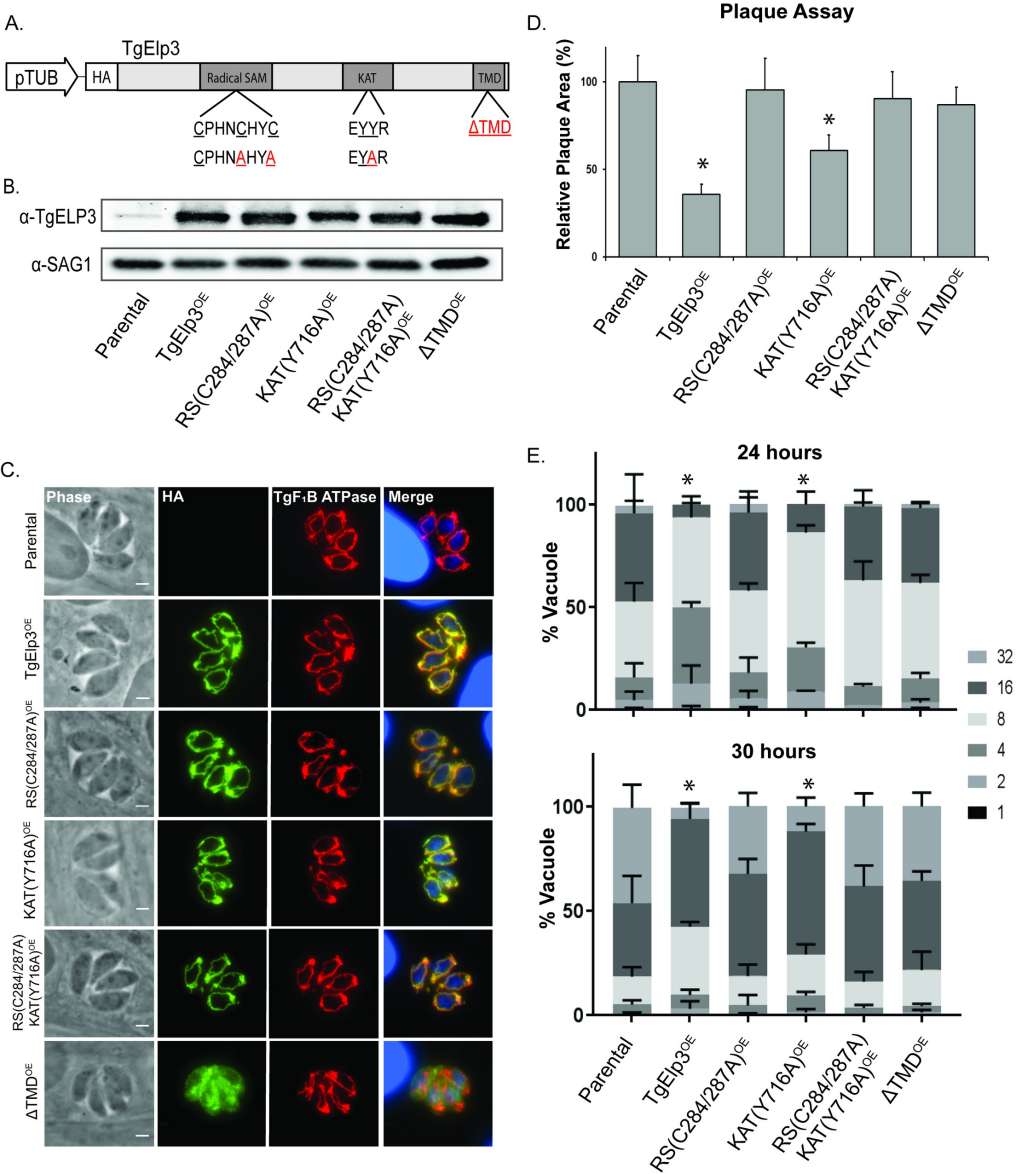
Overexpression of TgElp3 at the outer mitochondrial membrane in *Toxoplasma* causes a significant replication defect. (A) Schematic of the TgElp3^OE^ construct and various mutant forms. The constitutive tubulin promoter (pTub) was used to express full-length HA-tagged TgElp3 cDNA (cElp3). Letters in red represent mutated amino acids. (B) Western blot analysis of protein (100 μg) isolated from parental RHΔ*hx*, TgElp3^OE^ and mutant TgElp3^OE^ parasite strains using anti-TgELP3 and anti-SAG1 (loading control) antibodies. (C) IFA using anti-HA to detect TgElp3 (green) and anti-TgF_1_B ATPase as a known mitochondrial marker (red). DAPI (blue) was used as a co-stain to visualize the nuclei (note large nuclei are those of the host cells). Scale bar = 3μm. (D) Plaque assays to assess parasite proliferation. Plaque area was quantified using Image J; mean area of 30 plaques ± s.d. is depicted, *P<0.05 (two-way ANOVA). (E) Doubling assays to assess parasite replication rate. The number of parasites in 100 random vacuoles was quantified 24 and 30 hrs post-infection and the percentage of vacuoles containing the designated number of parasites ± s.d. is shown, *P<0.05 (two-way ANOVA).

Since we were unable to knockout or mutate endogenous TgElp3 and a recent CRISPR/Cas9 genetic screen identified TgElp3 as crucial for parasite growth (CRISPR score -3.28) [20], we pursued an overexpression strategy to gain insight into parasite processes affected by TgElp3. To ectopically overexpress ^HA^TgElp3, we generated an expression construct driven by the constitutively active *Toxoplasma* tubulin promoter. This construct was transfected into the RHΔ*hx* parasite strain and several independent clones were obtained. Overexpression of TgElp3 was confirmed by Western blot using antibody to TgElp3, and IFA confirmed its expected localization at the parasite OMM (Fig 2A–2C).

Upon initial observation, TgElp3 overexpressing parasites (TgElp3^OE^) appeared to grow slowly in culture. A plaque assay quantitatively confirmed a growth defect in the TgElp3^OE^ parasites compared to the parental line (Fig 2D). To further investigate this growth defect, we performed a doubling assay to assess parasite replication. Fig 2E shows that the replication rate of TgElp3^OE^ parasites is significantly slower than parental parasites. This replication defect was observed in multiple independent clones as well as type II ME49 parasites engineered to overexpress TgElp3 (S1 Fig).

We considered two possibilities why TgElp3 overexpression causes a replication defect: (1) independent of TgElp3 function, increased levels of protein at the OMM may negatively impact mitochondrial function, and (2) the replication defect is specific to the enzymatic activity of TgElp3 at the mitochondrion. To address these possibilities, we generated several mutant TgElp3^OE^ constructs: rSAM(C284A/C287A)^OE^, KAT(Y716A)^OE^ and a construct containing a premature stop codon to remove the transmembrane domain (ΔTMD^OE^). In addition, we generated a double mutant parasite line containing both rSAM and KAT mutations (rSAM(C284A/C287A)/KAT(Y716A)^OE^). For all parasite strains, protein expression was assessed by Western blot and localization by IFA using the anti-HA antibody (Fig 2A–2C). Mutations in the rSAM or KAT domains (or both) did not disrupt trafficking to the OMM, but as expected removal of the TMD did (Fig 2C). Given the fact that we cannot control for the number of ectopic gene copies or where they integrate within the genome, we isolated and performed the following experiments on at least two independent clonal parasite lines for each mutant construct to ensure results were in agreement.

Using the transgenic mutant TgElp3^OE^ parasite lines, we performed a plaque assay to assess parasite growth. Compared to parental parasites, the KAT(Y716A)^OE^ parasites grew significantly slower, similar to the unmodified TgElp3^OE^ parasites (Fig 2D). In contrast, parasites expressing a rSAM mutation or mislocalized TgElp3 (ΔTMD^OE^) grew at the same rate as parental parasites (Fig 2D). To assess parasite replication, doubling assays were performed. Consistent with the plaque assay results, a significant replication defect was detected in the unmodified TgElp3^OE^ and KAT(Y716A)^OE^ parasite lines at 24 and 30 hour time points (Fig 2E). Also consistent with the plaque assays, overexpression of TgElp3 harboring a rSAM mutation or mislocalized TgElp3^OE^ (ΔTMD^OE^) did not alter parasite replication. Considering that both the TgElp3 rSAM(C284A/C287A)^OE^ and double mutant rSAM(C284A/C287A)/KAT(Y716A)^OE^ parasite lines grew the same as parental, we conclude that the significant growth defect is not simply a result of too much protein at the OMM, but instead is due to increased levels of TgElp3. Moreover, since mislocalized overexpression of TgElp3 was tolerated with no detrimental effect on parasite growth, the replication defect in the TgElp3 overexpressing parasites seems directly linked to its function at the mitochondrion.

Overexpression of TgElp3 results in a significant replication defect whereas overexpression of a rSAM mutant TgElp3 is tolerated, suggesting this domain is essential for protein function. Despite mutating a functionally conserved amino acid previously reported as essential for Elp3 KAT activity in other species, we cannot rule out that this residue may function differently in *Toxoplasma*. To address this concern, a second TgElp3^OE^ KAT mutant parasite line was engineered by mutating two conserved tyrosine residues previously reported as essential for binding acetyl-coA to phenylalanine (KAT(Y715F/Y716F)^OE^) [17]. Phenotypic assays were performed and a significant replication defect similar to the KAT(Y716A)^OE^ parasite strain was observed [21].

In summary, overexpression of TgElp3 protein at the OMM significantly slows parasite replication. Normal growth rate is restored if the TMD is removed or if the rSAM domain is mutated, but not if the KAT domain is mutated. These results suggest that the rSAM domain of TgElp3 is critical for protein function, and confirms that TgElp3 activity is dependent on its localization to the OMM.

### Overexpression of TgRlmN at the OMM reduces parasite replication

We recently performed an analysis of the 59 predicted tail-anchored proteins present in the *Toxoplasma* genome and identified a second rSAM domain containing protein (TgRlmN) that also localizes to the parasite OMM like TgElp3 [9]. Similar to TgElp3, the CRISPR score for TgRlmN (-1.82) suggests that this gene is also integral for parasite growth [20]. In *E*. *coli*, RlmN is responsible for the 2-methlyadenosine (m^2^A) synthesis on purine 37 in the tRNA anticodon stem-loop and is required for translational accuracy [11]. Both TgElp3 and TgRlmN contain the canonical rSAM amino acid sequence motif (CxxxCxxC) that forms a [4Fe-4S] cluster required for methylation reactions. While these two proteins are both reported to modify tRNAs [5,11] and share a rSAM and TMD, the rest of the protein sequence is not well conserved. Moreover, TgRlmN is nearly half the size of TgElp3 (68 kDa versus 108 kDa, respectively) and lacks a lysine acetyltransferase domain.

**Fig 3:**
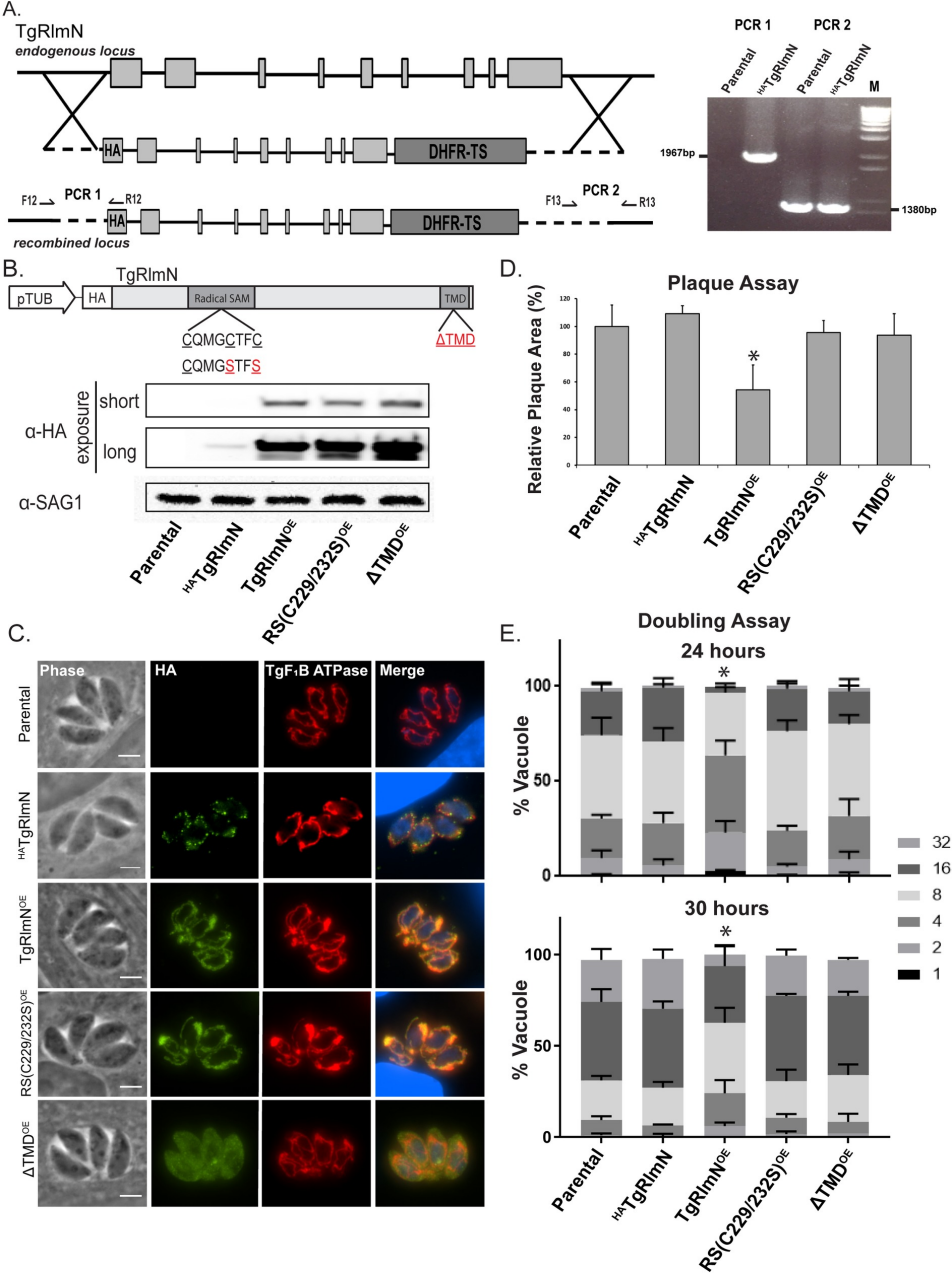
Overexpression of ^HA^TgRlmN at the OMM in *Toxoplasma* causes a significant replication defect. (A) Diagram of the construct used to replace the endogenous genomic TgRlmN locus by double homologous recombination. The replacement allele was identical except that it encoded an HA epitope tag fused in-frame at the N-terminus. Genomic PCR analysis confirmed correct replacement of the TgRlmN allele with the version that encodes ^HA^TgRlmN protein. The reverse primer for PCR1 is located within the HA-tag and PCR2 spans the 3’ recombination site. (B) Schematic of the construct used to ectopically overexpress TgRlmN and various mutant forms. The constitutive tubulin promoter (pTub) was used to express wild-type and mutant forms of TgRlmN cDNA; red letters show the amino acid mutations. Western blot analysis of protein (100 μg) isolated from parental (RHΔ*hx*Δ*ku80*), endogenously tagged (^HA^TgRlmN), and TgRlmN overexpressing (OE) parasite strains. Short exposure was sufficient to detect overexpressed versions of TgRlmN (upper panel), but overexposure was needed to detect the ^HA^TgRlmN driven by its native promoter (middle panel). SAG1 was analyzed as a loading control (lower panel). (C) IFA using anti-HA to detect TgRlmN (green) and anti-TgF_1_B ATPase (red) as a mitochondria marker, co-stained with DAPI (blue) to visualize the parasite nucleus. Scale bar = 3μm. (D) Parasite growth assessed by plaque assay. The mean area of 30 plaques ± s.d. was quantified using Image J, *P<0.05 (two-way ANOVA). (E) Parasite replication rate assessed at 24 and 30 hours by doubling assays. The percentage of vacuoles ± s.d. containing the designated number of parasites from 100 random vacuoles is shown, *P<0.05 (two-way ANOVA).

Since TgRlmN and TgElp3 reside at the same subcellular locale and possess similar protein characteristics, we hypothesized that overexpression of TgRlmN at the OMM will also cause a replication defect. To test this possibility, we substituted the endogenous TgRlmN genomic locus with an in-frame sequence to tag the N-terminus with an HA epitope (^HA^TgRlmN) in RHΔ*hx*Δ*ku80* parasites (Fig 3A). The allelic replacement was confirmed by genomic PCR (Fig 3A). Expression of the tagged ^HA^TgRlmN protein was assessed by Western blot, and IFA demonstrated its localization to the OMM (Fig 3B and 3C).

To determine whether altered TgRlmN levels produce a similar phenotype as seen for TgElp3, we overexpressed a wild-type (unmodified) TgRlmN (TgRlmN^OE^), a rSAM mutant (rSAM(C229/232S)^OE^), and a mislocalized TgRlmN (ΔTMD^OE^) driven by the constitutively active tubulin promoter. Overexpression of each version of ^HA^TgRlmN was confirmed by Western blot using anti-HA (Fig 3B). As seen for TgElp3, IFA revealed that the rSAM mutations did not disrupt trafficking to the OMM, but the removal of the C-terminal TMD did (Fig 3C).

We performed a plaque assay to assess parasite growth using the transgenic ^HA^TgRlmN parasite strains. Compared to the parental parasite strain, the TgRlmN^OE^ parasites grew significantly slower whereas the radical SAM mutant (rSAM(C229/232S)^OE^) and mislocalized TgRlmN (ΔTMD^OE^) parasite strains grew the same as parental (Fig 3D). In accordance with the observed growth defect, a significant replication defect was detected in the TgRlmN^OE^ parasite strain at 24 hr and 30 hr time points (Fig 3E). Mirroring our TgElp3 data, these results signify a functional role for the rSAM domain of TgRlmN and demonstrate that protein localization at the mitochondrion is important for function.

### Global translation is not altered by overexpression of TgElp3 or TgRlmN

The results presented above support the idea that the rSAM domain of TgElp3 and TgRlmN is critical for parasite growth. Both TgElp3 and TgRlmN contain the cysteine-rich rSAM domain (CxxxCxxC) that is required for RNA methylation reactions in other species. It was recently observed that the loss of wobble uridine mcm^5^ and mcm^5^s^2^ tRNA modifications in Elp3/Uba4 deficient *Saccharomyces cerevisiae* resulted in a decrease of global protein levels [12]. Similarly, loss of RlmN in *E*. *coli* increased misreading of the UAG stop codon, resulting in an error-prone phenotype and disrupted protein synthesis [11]. As these previous studies identified Elp3 and RlmN as important regulators of translation, we decided to assess protein synthesis in both of our overexpressing parasite strains (TgElp3^OE^ and TgRlmN^OE^) using two independent methods, polyribosome profiling and puromycin incorporation [22,23].

**Fig 4:**
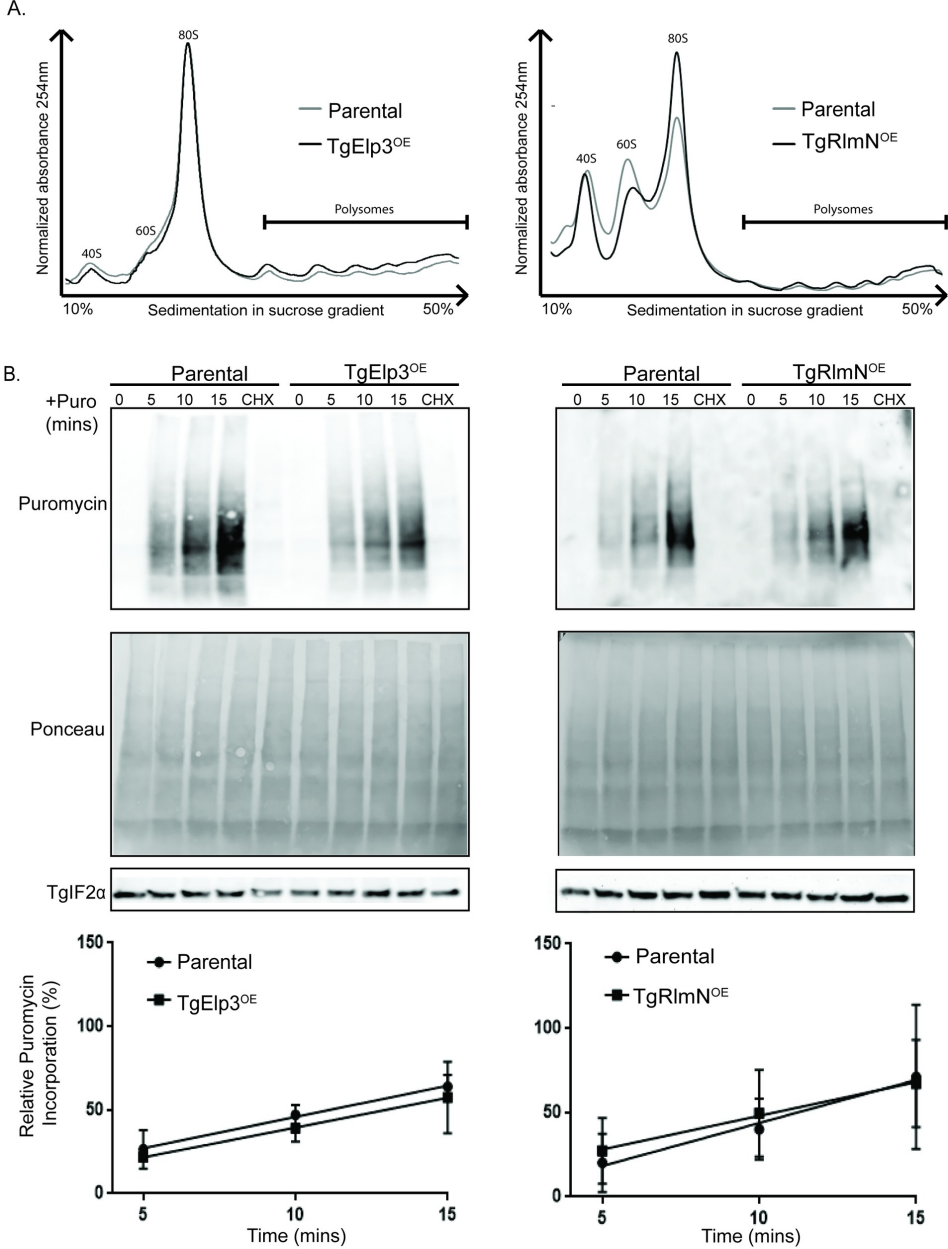
Overexpression of TgElp3 or TgRlmN does not affect global protein synthesis. (A) Equal amounts of lysate from TgElp3^OE^, TgRlmN^OE^, and the respective parental parasite strains were separated on a 10 to 50% sucrose gradient and polyribosome profiles were generated. (B) Freshly egressed parasites were incubated with 10 μg/mL puromycin (+Puro) for 5, 10, and 15 minutes (min). Control parasites were incubated with 100 μg/mL cycloheximide (CHX) for 10 min prior to a 15 min incubation with puromycin. At each time point, parasites were immediately lysed and 50 μg of protein was analyzed by Western blot using the anti-puromycin antibody. Ponceau S staining and probing with anti-TgIF2α show relatively equal loading and transfer. Representative blots of three independent assays are shown. Linear regression analysis showed no significant differences between the rate of puromycin incorporation between the parental and overexpressing parasite strains.

To evaluate the effect of TgElp3 or TgRlmN overexpression on global translation we first performed polyribosome profiling. This technique uses a sucrose gradient to separate mRNA-associated ribosome complexes [24]. Separation of these complexes is based on the number of associated ribosomes: free ribosome (small or large ribosome subunits), monosome (one ribosome residing on an mRNA), and polysome (multiple ribosomes residing on an mRNA). We treated freshly egressed parasites with cycloheximide to block the translocation step in protein synthesis, essentially “freezing” the ribosomal mRNA complexes. Parasite lysate was then subjected to polyribosome fractionation and analyzed by UV spectrometry. No overt differences were observed in the polysome profiles of parental parasites versus those overexpressing TgElp3 or TgRlmN (Fig 4A).

In addition to polyribosome profiling, which provides a snapshot of translation at the mRNA level, we performed a second method to assess translation at the protein level. This method uses puromycin, a tyrosyl-tRNA analog that is incorporated into nascent polypeptide chains. Puromycin incorporation inhibits further protein synthesis and results in C-terminally labeled proteins. To determine if protein synthesis is altered in TgElp3 or TgRlmN overexpressing parasite strains, we incubated freshly egressed parasites with puromycin for 5, 10, and 15 minutes; in addition, we used the protein synthesis inhibitor cycloheximide as a negative control. Western blot analysis using the anti-puromycin antibody revealed puromycin incorporation into *Toxoplasma* proteins in a time-dependent manner while cyclohexamide treatment blocked protein synthesis (Fig 4B). The amount of puromycin incorporation over time was used to assess translation rate for each parasite strain. No differences in puromycin incorporation were observed between the TgElp3 or TgRlmN overexpressing parasites and their respective parental controls (Fig 4B), suggesting that the fitness defect is not due to global perturbations in protein synthesis.

### The mcm^5^s^2^U tRNA modification is unchanged in TgElp3 overexpressing parasites

Very little is known regarding tRNA modifications in *Toxoplasma*. To date, only a single tRNA modification (pseudouridine) has been identified [25]. Given that Elp3 contains the canonical rSAM domain and is a tRNA modification enzyme in other species [26,27], we sought to determine if the Elp3-associated mcm^5^s^2^U tRNA modification exists in *Toxoplasma*.

**Fig 5:**
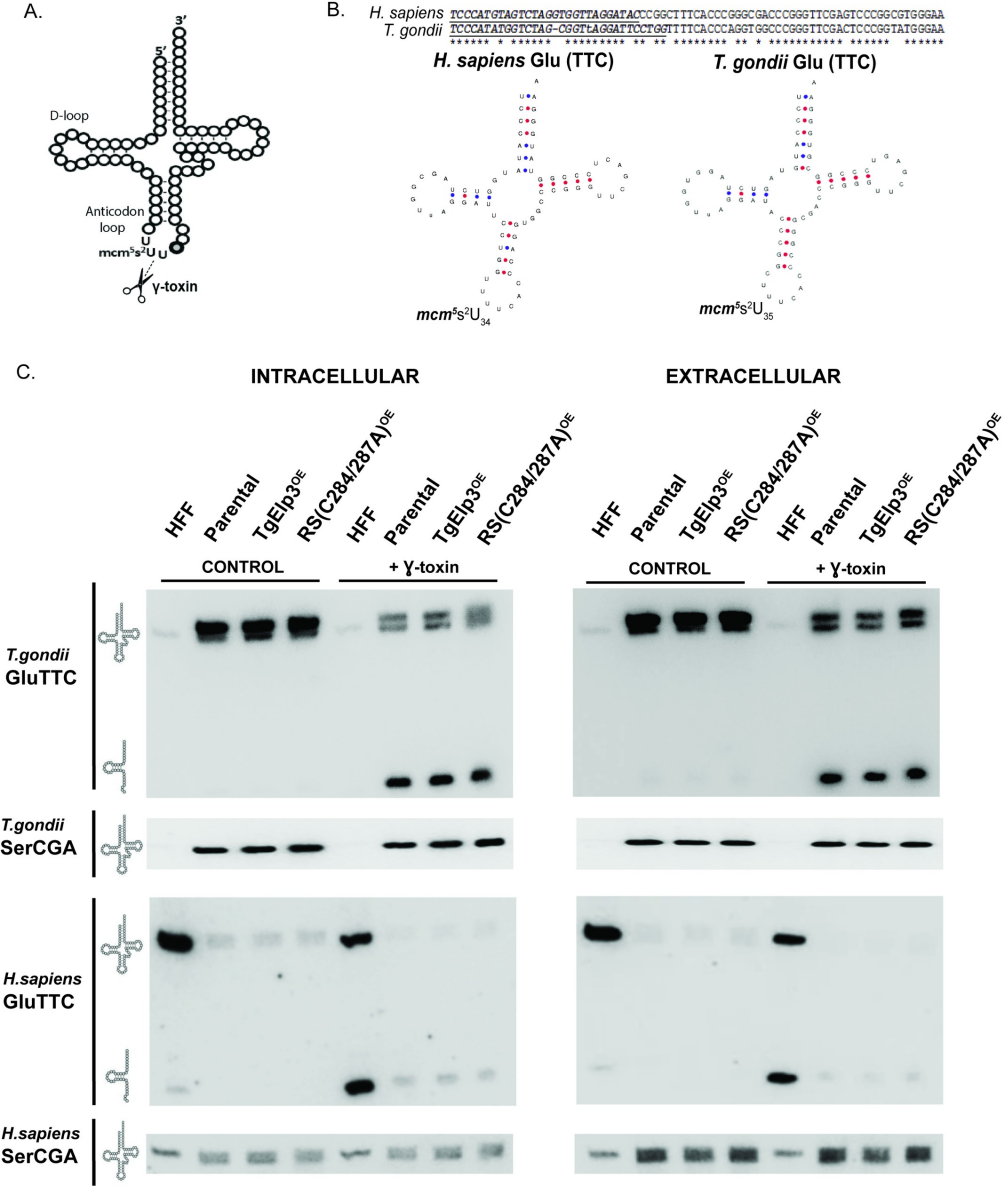
The tRNA^Glu^ mcm^5^s^2^ modification is present in *Toxoplasma*. (A) Depiction of where the γ-toxin endonuclease cleaves the mcm^5^s^2^U tRNA modification. Scissors represent cleavage site. (B) Sequence of the human (Hs-tRNA^Glu^) and *Toxoplasma* glutamic acid tRNAs (Tg-tRNA^Glu^); underlined and italicized sequences were used for Northern blot probe design. Predicted Hs-tRNA^Glu^ and Tg-tRNA^Glu^ structures generated by tRNAscan-SE [35,36]. (C) Northern blot of 5 μg total RNA isolated from intracellular and extracellular parental, TgElp3^OE^, and rSAM(C284A/C287A)^OE^ parasites, as well as uninfected human foreskin fibroblast cells (HFF), incubated with or without the γ-toxin endonuclease for 10 min at 30°C. GluTTC and SerCGA probes specific to *T*. *gondii* and *H*. *sapiens* were used. The probe used for each blot is listed on the left along with an image representative of intact or cleaved tRNA.

Previous studies have shown that the loss of Elp3 in *S*. *cerevisiae* correlates with the loss of the mcm^5^ side-chain, generating resistance to the *Kluyveromyces lactis* killer toxin (γ-toxin) [5,28,29]. This γ-toxin is a tRNA endonuclease that cleaves tRNA at the 3’ end of the wobble nucleoside mcm^5^s^2^U [30] (Fig 5A). The mcm^5^s^2^U tRNA modification identified in *S*. *cerevisiae* is only present in lysine, glutamic acid, and glutamine tRNAs, and its formation requires two enzymes: (1) Elp3 for the formation of mcm^5^ modification, and (2) cytosolic thiouridylase (Ctu1/Ctu2) for the formation of the 2-thio (s^2^) group [26,27,31–33]. Blast analyses of the *Toxoplasma* proteome using *S*. *cerevisiae* Ctu1/Ctu2 sequences identified two putative cytosolic thiouridylase proteins, TGME49_309020 and TGME49_294380 (ToxoDB v.9.0, [34]), suggesting that the mcm^5^s^2^U tRNA modification may exist in *Toxoplasma*.

To perform the γ-toxin *in vitro* enzymatic assay, we used total RNA samples isolated from parental, TgElp3^OE^, and rSAM(C284A/C287A)^OE^ parasite lines, along with an uninfected human foreskin fibroblast (HFF) control sample, and incubated them with recombinant γ-toxin or an empty vehicle control. To assess cleavage, we used Northern blot probes specific to *Toxoplasma* glutamic acid tRNA (Tg-tRNA^Glu^) as well as a control probe for serine tRNA (Tg-tRNA^Ser^), which does not possess the mcm^5^s^2^U tRNA modification. Since *Toxoplasma* is an intracellular pathogen, host cell contamination is possible, so we used RNA extracted from both intracellular and extracellular tachyzoites, and included Northern blot probes specific to human glutamic acid (Hs-tRNA^Glu^) and serine tRNAs (Hs-tRNA^Ser^). Interestingly, Tg-tRNA^Glu^ contains an extra nucleotide in the D-loop, making it 73 bp long compared to the Hs-tRNA^Glu^ composed of 72 bp. This extra base pair makes the *Toxoplasma* wobble uridine base pair 35, not the typical 34 (Fig 5B).

Upon treatment with γ-toxin, we observed cleavage of the Tg-tRNA^Glu^, demonstrating that this mcm^5^s^2^U tRNA modification is present in *Toxoplasma* (Fig 5C). A similar pattern was observed in the HFF control sample when probed with anti-Hs-tRNA^Glu^ (Fig 5C). As expected, there was no cleavage of Tg-tRNA^Ser^ or Hs-tRNA^Ser^ with treatment of γ-toxin (Fig 5C). Despite efforts to design probes specific to either human or *Toxoplasma*, we observed some non-specific binding of the Hs-tRNA^Ser^ probe to the *Toxoplasma* RNA; however, the Tg-tRNA^Ser^ appears specific to *Toxoplasma* (Fig 5C).

Unexpectedly, a doublet was observed in the *Toxoplasma* samples when using the Tg-tRNA^Glu^ probe (Fig 5C). Initially, we thought this may be due to host cell contamination; however, when using the Hs-tRNA^Glu^ probe against the *Toxoplasma* samples, we did not detect Hs-tRNA^Glu^ (Fig 5C). Non-specific binding of the Tg-tRNA^Glu^ probe to another *Toxoplasma* tRNA could explain the doublet. The *Toxoplasma* aspartic acid tRNA (Tg-tRNA^Asp^) shares the closest sequence homology, matching 17 of the 28 nucleotides in the Tg-tRNA^Glu^ probe. Despite some sequence similarity between the Tg-tRNA^Glu^ and Tg-tRNA^Asp^ sequences, there is a one-third mismatch between Tg-tRNA^Glu^ probe and the Tg-tRNA^Asp^ sequence, making it unlikely to be the cause of the doublet. Another possibility is that there may be a portion of Tg-tRNA^Glu^ that has some unresolved secondary structure causing a band shift in the gel. Curiously, a doublet has also been observed in *S*. *cerevisiae* when probed for the glutamic acid tRNA [37], but an explanation remains unknown. Nevertheless, there is no difference in the levels of intact or cleaved Tg-tRNA^Glu^ levels between the parental, TgElp3^OE^, and rSAM(C284A/C287A)^OE^ samples, indicating that the fitness defect in the TgElp3 overexpressing parasites is not due to excessive mcm^5^s^2^U tRNA^Glu^ modifications (Fig 5C).

## Discussion

This study is the first to explore the functional significance of two mitochondrial tail-anchored rSAM domain containing proteins, TgElp3 and TgRlmN, in an early-branching eukaryote of medical importance. In these studies, we determined that overexpression of TgElp3 or TgRlmN at the parasite mitochondrion results in a significant replication defect, but overexpression of these proteins bearing a mutant rSAM domain or lacking their TMD is well-tolerated and does not impair fitness. These findings highlight the importance of TgElp3 and TgRlmN as being intimately connected to their presence at the OMM, and confirm the essential nature of the rSAM domain in their biological activities. In other species, the rSAM domain of Elp3 and RlmN is responsible for post-transcriptional tRNA modifications, specifically the mcm^5^ and ncm^5^ wobble uridine modifications and methylation of adenosine 37, respectively. These tRNA modifications are thought to alter protein synthesis in a context dependent manner, but the exact mechanisms are not well understood [38–41].

In a *S*. *cerevisiae Elp3/Uba4* knockout strain, the loss of the mcm^5^s^2^ tRNA modification is associated with drastically decreased protein levels as determined by Coomassie blue staining, but protein levels were partially restored with overexpression of tRNA^Lys^ [12]. In a subsequent study, ribosomal footprinting identified an accumulation of ribosomes at the GAA and CAA codons in a knockout Elp3 yeast strain, suggesting that these codons were translated less efficiently by hypomodified tRNAs; however, polyribosome gradient profiles of the mcm^5^s^2^ deficient strains were indistinguishable from wild-type, suggesting that polyribosome profiling may not be sensitive enough to identify small alterations in global protein synthesis [42]. Since initiation is typically the rate-limiting step of eukaryotic translation, and tRNA modifications predominantly affect translation elongation, changes at the elongation step may be missed by global protein synthesis assays [43,44]. In accordance, we did not detect significant differences between the TgElp3 or TgRlmN overexpression and parental parasites using polyribosomal profiling or puromycin incorporation. These methods are limited in that they are unlikely to identify small changes in protein synthesis, and codon-specific translation defects may only affect a subset of genes required for a particular cellular process. Additional study is required before definitively concluding that overexpression of TgElp3 or TgRlmN does not alter translation on a subset of mRNA transcripts.

In this study, we established that the puromycin incorporation can be applied to assess protein synthesis in *Toxoplasma*. This technique can likely be adapted to other apicomplexan parasites including *Plasmodium spp*. and *Cryptosporidium spp*., which adds a valuable new tool for studying protein synthesis in these protozoa.

Using the *in vitro* γ-toxin assay, we identified a second tRNA modification in *Toxoplasma*, tRNA^Glu^ mcm^5^s^2^U35. This wobble uridine modification is predicted to change the physical interaction between the anticodon and codon with the mcm^5^ modification preferentially translating–G ending codons while the mcm^5^s^2^ favors–A ending codons [45,46]. In these studies, we were unable to attribute the TgElp3^OE^ replication defect to changes in the tRNA^Glu^ mcm^5^s^2^U35 modification levels. Knowing that the tRNA^Glu^ mcm^5^s^2^U35 modification requires a second enzyme (Ctu1/Ctu2), and that Elp3 is known to modify 11 different tRNAs in other organisms [5,37], we cannot rule out the possibility that overexpression of TgElp3 is causing hypermodification of tRNAs and subsequent slowed parasite growth. Since we only assessed changes in the Elp3 putative downstream modification mcm^5^s^2^U35 on a single tRNA^Glu^, more studies are needed to quantitatively assess the modification status of each tRNA.

While Elp3 is well-conserved across all domains of life, RlmN-like genes are only present in bacteria, plants, and algae [6,10]; however, here we report the first RlmN-like gene identified in a protozoa. Despite sequence conservation of Elp3 in *Toxoplasma*, homologues to the other five components of the Elongator complex in which Elp3 typically associates with are missing [2]. Even more intriguing is the localization of TgElp3 and TgRlmN to OMM via a TMD positioned with the catalytic domains protruding into the cytosol [9]. This TMD is only present in the phylum Apicomplexa and few select species within the Chromalveolata supergroup, suggesting these rSAM enzymes may have an alternative function that is linked to the mitochondrion in these species.

One possible reason for this peculiar localization is that specific tRNAs may require modification in order to be imported into the parasite mitochondrion. Due to the complete lack of tRNA genes in the mitochondrial genome, *Toxoplasma* must import all tRNAs required for mitochondrial translation [47]. Several reports suggest that tRNA modifications could regulate which tRNAs are imported into the mitochondria and which remain in the cytosol [48,49]. For example, in *Leishmania tarentolae* nuclear encoded tRNAs containing the mcm^5^ modification are found mostly in the mitochondria; in contrast, cytosolic tRNAs contain both the mcm^5^ and s^2^ modifications (mcm^5^s^2^), indicating that the cytosol specific 2-thiolation could play an inhibitory role in mitochondrial tRNA import [48].

## Materials and methods

### Plasmid DNA construction

For all constructs, the In-Fusion HD cloning kit was used to insert DNA fragments into plasmid backbones (Clontech #011614). Primers for each of the constructs are listed in S1 Table. The Phusion High Fidelity DNA Polymerase (Thermo Fisher #F530L) was used for all PCR amplification reactions. Details specific to each plasmid are below.

#### Allelic replacement plasmids

To generate the TgElp3 allelic replacement plasmids the upstream promoter region (~1,500bp) of TgElp3 was amplified from RHΔ*hx*Δ*ku80* genomic DNA using primers F3/R3, and ^HA^TgElp3 cDNA was amplified using primers F4/R4 from the previously published construct: TubHX-^HA^TgElp3 [2]. These two PCR products were combined and used as template for a “stitching PCR” reaction using primers F3/R4 to create the TgElp3prom+ ^HA^TgElp3cDNA PCR product. Next, the downstream region (~1,500bp) of TgElp3 was amplified from RHΔ*hx*Δ*ku80* genomic DNA using primers F5 and R5. The TgElp3 downstream region amplicon was inserted into the HindIII restriction site of the pDHFR-TS plasmid (pDHFR-TS-dsElp3) [50,51]. This plasmid (pDHFR-TS-dsElp3) was then digested using DraIII and the TgElp3prom+ ^HA^TgElp3cDNA PCR amplicon was inserted to create the ^HA^TgElp3 endogenous replacement construct (TgElp3-pDHFR-TS).

The QuickChange II XL Site-Directed Mutagenesis kit (Agilent Technologies. #200521) was used to generate the mutant constructs: ^HA^TgElp3-rSAMmut(C284A) and ^HA^TgElp3-KATmut(Y715/716A), using primers F6/R6 and F7/R7, respectively. Constructs were sequenced using primers F8 and R8. Prior to transfection, plasmids were linearized using the restriction enzyme SpeI. Parasites were screened for correct genomic integration using primers F1/R1 and F2/R2.

To generate the TgRlmN allelic replacement vector, the TgRlmN gene genomic DNA (7,204 bp; F9/R9), promoter (1,817 bp; F10/R10) and downstream (3,315 bp; F11/R11) regions were amplified from RHΔ*hx*Δ*ku80* genomic DNA. The TgRlmN promoter was inserted into the HindIII site of the pDHFR-TS plasmid [50]. To N-terminally tag the TgRlmN gene the genomic DNA amplicon (7,204 bp) was first subcloned into the EcorV site of the previously published TubHA tagging vector [9]. The HA-TgRlmN gene was then amplified from the subcloning vector (F14/R14) and inserted into the EcorV site immediately downstream of the TgRlmN promoter in the pDHFR-TS backbone. The downstream amplicon (3,315 bp) was inserted into the XbaI restriction site to generate the TgRlmN allelic replacement vector (TgRlmN-pDHFR-TS). This construct was sequenced using the M13 forward and reverse primers. Prior to transfection, the construct was linearized using the restriction enzyme NcoI. Parasites were screened for correct genomic integration using F12/R12 and F13/R13.

#### Overexpression plasmids

To generate the overexpression mutant constructs, the previously published wild-type TgElp3^OE^ construct [2] was used. The QuickChange II XL Site-Directed Mutagenesis kit (Agilent Technologies. #200521) was used to generate the following mutant constructs: rSAM(C284/C287A)^OE^ (F15/R15 & F16/R16), KAT(Y716A)^OE^ (F17/R17) and ΔTMD^OE^ (F18/R18). Site-Directed mutagenesis was performed multiple times using different primers to generate both cysteine mutations in the rSAM domain and for the KAT + rSAM mutant. The plasmid sequences were confirmed using primers F23 and R23. Prior to transfection each plasmid was linearized using the NotI restriction enzyme.

To overexpress TgElp3 in the type II ME49 parasite strain, wild-type TgElp3 was amplified from the ^HA^TgElp3^OE^ construct and inserted into the HindIII restriction site of the pDHFR plasmid using primers F19/R19.

To generate the TgRlmN overexpression constructs, TgRlmN cDNA was amplified from the previously published TubHA-RlmN-DHFR3'UTR-pDHFR-TS plasmid [9] using F20/R20 primers or the F20/R20b primers to remove the TgRlmN TMD. These amplicons were inserted into the BglII and EcorV restriction sites within the TubHX cloning vector [51]. The QuickChange II XL Site-Directed Mutagenesis kit (Agilent Technologies #200521) was used with primers F22 and R22 to generate the TgRlmN rSAM(C229/232S)^OE^ mutant construct. The plasmid sequences were verified using primers F23 and R23. The NotI enzyme was used to linearize the TgRlmN overexpression plasmids prior to transfection.

#### Parasite maintenance and generation of transgenic parasites

For all experiments, *Toxoplasma* was cultured in human foreskin fibroblasts (HFF). The HFF cell line is commercially available from: ATCC, HFF-1 SCRC-1041™. HFFs were cultured with Dulbecco’s Modified Eagle’ Medium (DMEM) supplemented with 10% heat-inactivated fetal bovine serum (FBS) at 37°C and 5% CO_2_. The HFF medium was changed to DMEM supplemented with 1% FBS prior to parasite inoculation.

To express various plasmids ectopically or to modify the endogenous locus, freshly egressed *Toxoplasma* tachyzoites (RHΔ*hx*Δ*ku80*, RHΔ*hx*, or ME49 as described in the text) were electroporated with 75 μg of linearized DNA. A pmini-Ku80 plasmid (Chunlin Yang and Gustavo Arrizabalaga, unpublished) was simultaneously transfected with each ectopic overexpression construct into the ^HA^TgRlmN parasite line (RHΔ*hx*Δ*ku80* background) to facilitate genomic integration using non-homologous end joining. Following transfection, parasites were split evenly between multiple flasks containing confluent HFF monolayers to isolate independent clones. Drugs used for selection were added 24 hours post-transfection and parasites were maintained under selection for a minimum of three passages prior to isolation of individual clones. To select for parasites containing overexpression plasmids (HXGPRT gene), 25 μg/mL of mycophenolic acid (MPA) and 50 μg/mL of xanthine (XAN) was used for selection [52]. For the allelic replacement plasmids containing the DHFR-TS gene, 1 μM pyrimethamine selection was used [50]. Limiting dilution was used to isolate individual clones from populations of transfected parasites.

### Western blot analysis

Parasites from a freshly lysed T-25 cm^2^ flask (~3 x 10^7^ parasites) were pelleted and washed once in ice-cold phosphate buffered saline (PBS) followed by lysis in RIPA buffer supplemented with cOmpleteTM, Mini, EDTA-free protease inhibitor (Roche #11836170001) and sonicated three times on ice for 10 seconds with a microtip sonicator. Insoluble material was removed by centrifugation at 21,000 x g for 10 min at 4°C and cleared lysate was quantified using a detergent compatible (DC^TM^) protein assay kit (Bio-Rad #5000111). For each sample, equal amounts of protein were mixed with Laemmli sample buffer supplemented with 5% beta-mercaptoethanol and heated at 95°C for 10 min. Samples were centrifuged at 21,000 x g for 3 min and subjected to SDS-PAGE with precast 4 to 20% Mini-PROTEAN TGX gels (Bio-Rad #4568094). The Transblot SD semidry transfer system was used to transfer proteins to a nitrocellulose membrane (Bio-Rad #1703940). Blots were subjected to Ponceau S staining to ensure relatively equal protein loading and transfer. Membranes were then washed with Tris buffered saline-Tween 20 (TBST) and blocked with 5% milk-TBST for 30 min. Membranes were incubated with primary antibodies at 4°C overnight. Membranes were washed 3 x 10 min in TBST followed by incubation with secondary HRP-linked antibodies for 1 hour at room temperature. Membranes were washed for 3 x 10 min and proteins were detected with SuperSignal^TM^ West Femto Maximum Sensitivity Substrate (Thermo Fisher #34094) and imaged on a FluorChem R imager (Bio-Techne).

The following primary antibodies were used at the dilutions indicated: rat anti-HA (1:2,000, Roche #11867423001), rabbit anti-TgELP3 (1:2,000) [14], mouse anti-SAG1 (1:2000, Genway #MA1-83499), mouse anti-TgF_1_B ATPase (1:4,000) [15,16], and mouse anti-puromycin clone 12D10 (1:2,000, Sigma Aldrich #MABE343). Secondary HRP-linked antibodies for Western blot analysis included donkey anti-rabbit (1:2,000, GE Healthcare #NA934), sheep anti-mouse (1:5,000, GE Healthcare #NA931) and goat-anti rat (1:2,000, GE Healthcare #NA935).

### Immunofluorescence assays

Freshly lysed parasites were inoculated onto confluent HFF monolayers grown on coverslips in a 24-well plate. Plates were fixed with 4% paraformaldehyde in PBS and blocked for 30 min in 3% bovine serum albumin (BSA) in PBS. Cells were permeabilized with 0.2% Triton X-100 in BSA-PBS for 10 min. All antibodies were diluted in 3% BSA-PBS and were applied in sequential order. Cells were incubated with rat anti-HA (1:2,000, Roche #11867423001) for 1 hour at room temperature followed by 3 x 10 min PBS washes and 1 hour incubation with anti-rat Alexa Fluor 488 (1:5,000, Thermo Fisher #A-11006) at room temperature. Cells were washed 3 x 10 min with PBS followed by overnight incubation with mouse anti-TgF_1_B ATPase (1:4,000) [15,16] at 4°C. Cells were washed 3 x 10 min followed by 1 hour incubation with anti-mouse Alexa Fluor 594 (1:5,000, Thermo Fisher A-11005) at room temperature. Coverslips were washed 3 x 10 min with PBS. The nucleic acid stain 4’, 6-diamidino-2-phenylindole dihydrochloride (DAPI, Life Technologies #D1306) diluted 1:1,000 in PBS was applied for 10 min at room temperature and then washed 3 x 10 min at room temperature. Coverslips were mounted using Vectashield antifade mounting medium (Vector Labs #H-1000).

### Parasite growth assays

Plaque assays were used to assess parasite growth [53,54]). Intracellular parasites were harvested from the host cell monolayer by scraping, syringe lysing (25-guage needle) and filtering through a 3.0 μm polycarbonate filter. Parasites were counted using a hemocytometer and 500 parasites were inoculated into a single well of a 12-well plate containing confluent HFFs. For each assay, an individual parasite line was plated in triplicate. Four hours after inoculation, uninvaded parasites were removed and fresh medium was replaced. The cultures were incubated undisturbed for 6 days and then fixed with 100% ice-cold methanol. To visualize plaques, plates were stained with crystal violet (Sigma-Aldrich #C38886). Pictures of individual plaques were taken at random using a Leica inverted DM16000B microscope (40x dry objective). The mean area of 30 plaques was measured using ImageJ for each of three independent experiments. The plaque area was analyzed using two-way analysis of variance (ANOVA) using GraphPad Prism version 7.03 for Windows, GraphPad Software, La Jolla California USA, http://www.graphpad.com.

Doubling assays were used to assess parasite replication [55,56]. Intracellular parasites were harvested as described above; 150,000 parasites were used to inoculate a HFF monolayer in a 6-well plate and after 4 hours the medium was changed to remove any uninvaded parasites. Plates were fixed at 24 and 30 (or 48 for ME49) hours post inoculation using ice-cold methanol. The number of parasites in 100 random vacuoles were counted for each parasite strain and the average number of parasites per vacuole was calculated; this assay was repeated three times. The average number of parasites per vacuole for each parasite line was analyzed using two-way ANOVA using GraphPad Prism version 7.03 for Windows, GraphPad Software, La Jolla California USA, http://www.graphpad.com.

### Polyribosome analysis

For polyribosome analysis, one T-175 cm^2^ flasks of freshly egressed parasites were treated with 50 μg/mL of cycloheximide for 10 min [57,58]. Parasites were pelleted by centrifugation at 1,500 x g for 10 min at 4°C. Pelleted parasites were resuspended in 1 mL of ice-cold PBS containing 50 μg/mL cyclohexamide and passed through a 25-guage needle to remove any residual host cell debris. Parasites were then washed three times with 10 mL of ice-cold PBS containing 50 μg/mL of cycloheximide. Parasites were pelleted by centrifugation at 1000 x g for 10 min at 4°C and re-suspended in 500 μL of lysis buffer (20 mM Tris-HCL (pH 7.9), 100 mM NaCl, 10 mM MgCl_2_, 0.4% NP-40, 50 μg/mL cycloheximide). Parasites were passed through a 25-guage needle several times to facilitate parasite lysis followed by a 10 min incubation on ice. Insoluble proteins were cleared by centrifugation at 21,000 x g for 10 min at 4°C and 400 μL of lysate was layered onto 10 to 50% sucrose gradients prepared in lysis buffer without detergent. Polyribosome complexes were resolved by centrifugation using a Beckman Coulter SW41Ti rotor at 40,000 rpm at 4°C for 2 hr. Gradients were fractionated by a BioComp Instruments gradient station and the absorbance was measured at 254nm.

### Puromycin incorporation

To determine if the puromycin incorporation could be used to assess protein synthesis in *Toxoplasma*, we assessed the incorporation of puromycin in freshly egressed parasites. Approximately 2.5x10^6^ parasites were incubated with 10 μg/mL puromycin (SIGMA #P8833) for 5, 10 and 15 min at 37°C. The control sample was treated with 100 μg/mL of cycloheximide for 10 min at 37°C prior to the addition of puromycin. Cells were pelleted and washed once in ice-cold PBS followed by Western blot analysis as described above. Protein was quantified by densitometry using Image J [59]. Puromycin incorporation was normalized to the Ponceau S staining and the rate of translation was calculated over time. Slopes from three independent experiments were calculated and analyzed by linear regression using GraphPad Prism version 7.03 for Windows, GraphPad Software, La Jolla California USA, http://www.graphpad.com.

### γ-toxin assay

Total RNA was extracted from both extracellular and intracellular parasites. For intracellular parasites two T-175 cm^2^ flasks were scraped, syringe lysed using a 25-guage needle and passed through a 3.0 μm polycarbonate filter (Whatman) to remove host cell debris and washed three times in PBS. After the final wash, parasites were resuspended in 250 μL of PBS and 750 μL of TRIzol^TM^ LS Reagent (Invitrogen) and RNA was isolated according to the manufacturer’s protocol. For the extracellular parasite sample, two T-175 cm^2^ flasks of freshly egressed parasites were filtered and further processed the same as the intracellular samples. For a control, RNA was also isolated from human foreskin fibroblast cells (parasite host cell). The final RNA pellet was suspended in 30 μL of DEPC treated water and the RNA concentration was quantified using a Nanodrop. To check the quality of the total RNA and amount of host cell contamination, 3 μg of RNA was run on a 0.8% agarose gel.

For each sample, 5 μg of total RNA was mixed with purified γ-toxin protein in 10 mM Tris-HCl (pH 7.5), 10 mM MgCl_2_, 50 mM NaCl and 1mM dithiothreitol (pH 7.5) and incubated for 10 min at 30°C. The samples were separated on a 10% polyacrylamide, 7 M urea gel, and transferred to an Amersham Hybond-XL membrane (GE Healthcare). Oligonucleotides used to detect tRNAs were 5’- GTATCCTAACCACCTAGACTACATGGGA-3’ (Tg-tRNA^Glu^), 5’- TCTCCTTAACCACTCGGACACA-3’ (Tg-tRNA^Ser^), 5’-CCAGGAATCCTAACCGCTAGACCATATGGGA-3’ (Hs-tRNA^Glu^) and 5’- GCCTTAACCACTCGGCCATCACAGC-3’ (Hs-tRNA^Ser^). Oligonucleotides were labeled by using adenosine [γ^32^P]-triphosphate (6000 Ci/mmol, Amersham Biosciences) and polynucleotide kinase (NEB). Northern blots were visualized by a Phosphor-Imager and quantified using ImageJ [59].

## Supporting information

S1 Fig Overexpression of TgElp3 at the outer mitochondrial membrane in a *Toxoplasma* type II strain causes a significant replication defect. Replication rate was assessed in the parental ME49 and two independent TgElp3 overexpressing ME49 parasite strains (TgElp3^OE^ C1 and C2). Doubling assays were performed at 24 and 48 hours; the number of parasites in 100 random vacuoles was quantified and the percentage of vacuoles containing the designated number of parasites ± s.d. is shown, *P<0.05 (two-way ANOVA).
(TIF)

S1 Table List of primers used in this study. (TIF)

## References

[1] Kimura S, Suzuki T. Iron-sulfur proteins responsible for RNA modifications. Biochim Biophys Acta. 2015;1853: 1272–1283. doi: 10.1016/j.bbamcr.2014.12.010 25533083 10.1016/j.bbamcr.2014.12.010

[2] Stilger KL, Sullivan WJ. Elongator protein 3 (Elp3) lysine acetyltransferase is a tail-anchored mitochondrial protein in Toxoplasma gondii. J Biol Chem. 2013;288: 25318–25329. doi: 10.1074/jbc.M113.491373 23878194 10.1074/jbc.M113.491373 PMC3757196

[3] Otero G, Fellows J, Li Y, de Bizemont T, Dirac AM, Gustafsson CM, et al Elongator, a multisubunit component of a novel RNA polymerase II holoenzyme for transcriptional elongation. Mol Cell. 1999;3: 109–118. 10024884 10.1016/s1097-2765(00)80179-3

[4] Wittschieben BO, Otero G, de Bizemont T, Fellows J, Erdjument-Bromage H, Ohba R, et al A novel histone acetyltransferase is an integral subunit of elongating RNA polymerase II holoenzyme. Mol Cell. 1999;4: 123–128. 10445034 10.1016/s1097-2765(00)80194-x

[5] Huang B, Johansson MJO, Byström AS. An early step in wobble uridine tRNA modification requires the Elongator complex. RNA. 2005;11: 424–436. doi: 10.1261/rna.7247705 15769872 10.1261/rna.7247705 PMC1370732

[6] Selvadurai K, Wang P, Seimetz J, Huang RH. Archaeal Elp3 catalyzes tRNA wobble uridine modification at C5 via a radical mechanism. Nat Chem Biol. 2014;10: 810–812. doi: 10.1038/nchembio.1610 25151136 10.1038/nchembio.1610 PMC4479141

[7] Rezgui VAN, Tyagi K, Ranjan N, Konevega AL, Mittelstaet J, Rodnina MV, et al tRNA tKUUU, tQUUG, and tEUUC wobble position modifications fine-tune protein translation by promoting ribosome A-site binding. Proc Natl Acad Sci USA. 2013;110: 12289–12294. doi: 10.1073/pnas.1300781110 23836657 10.1073/pnas.1300781110 PMC3725067

[8] Toh S-M, Xiong L, Bae T, Mankin AS. The methyltransferase YfgB/RlmN is responsible for modification of adenosine 2503 in 23S rRNA. RNA. 2008;14: 98–106. doi: 10.1261/rna.814408 18025251 10.1261/rna.814408 PMC2151032

[9] Padgett LR, Arrizabalaga G, Sullivan WJ. Targeting of tail-anchored membrane proteins to subcellular organelles in Toxoplasma gondii. Traffic. 2017;18: 149–158. doi: 10.1111/tra.12464 27991712 10.1111/tra.12464 PMC5325807

[10] Atkinson GC, Hansen LH, Tenson T, Rasmussen A, Kirpekar F, Vester B. Distinction between the Cfr methyltransferase conferring antibiotic resistance and the housekeeping RlmN methyltransferase. Antimicrob Agents Chemother. 2013;57: 4019–4026. doi: 10.1128/AAC.00448-13 23752511 10.1128/AAC.00448-13 PMC3719738

[11] Benítez-Páez A, Villarroya M, Armengod M-E. The Escherichia coli RlmN methyltransferase is a dual-specificity enzyme that modifies both rRNA and tRNA and controls translational accuracy. RNA. 2012;18: 1783–1795. doi: 10.1261/rna.033266.112 22891362 10.1261/rna.033266.112 PMC3446703

[12] Klassen R, Grunewald P, Thüring KL, Eichler C, Helm M, Schaffrath R. Loss of anticodon wobble uridine modifications affects tRNA(Lys) function and protein levels in Saccharomyces cerevisiae. PLoS ONE. 2015;10: e0119261 doi: 10.1371/journal.pone.0119261 25747122 10.1371/journal.pone.0119261 PMC4352028

[13] Schwalm EL, Grove TL, Booker SJ, Boal AK. Crystallographic capture of a radical S-adenosylmethionine enzyme in the act of modifying tRNA. Science. 2016;352: 309–312. doi: 10.1126/science.aad5367 27081063 10.1126/science.aad5367 PMC5629962

[14] Stilger KL. Identification of TgElp3 as an essential, tail-anchored mitochondrial lysine acetyltransferase in the protozoan pathogen toxoplasma gondii. [Internet]. Indiana University School of Medicine. 2014 Available: https://scholarworks.iupui.edu/handle/1805/4660

[15] Lavine MD, Arrizabalaga G. Analysis of monensin sensitivity in Toxoplasma gondii reveals autophagy as a mechanism for drug induced death. PLoS ONE. 2012;7: e42107 doi: 10.1371/journal.pone.0042107 22848721 10.1371/journal.pone.0042107 PMC3405052

[16] Beck JR, Chen AL, Kim EW, Bradley PJ. RON5 is critical for organization and function of the Toxoplasma moving junction complex. PLoS Pathog. 2014;10: e1004025 doi: 10.1371/journal.ppat.1004025 24651769 10.1371/journal.ppat.1004025 PMC3961375

[17] Wittschieben BO, Fellows J, Du W, Stillman DJ, Svejstrup JQ. Overlapping roles for the histone acetyltransferase activities of SAGA and elongator in vivo. EMBO J. 2000;19: 3060–3068. doi: 10.1093/emboj/19.12.3060 10856249 10.1093/emboj/19.12.3060 PMC203375

[18] Okada Y, Yamagata K, Hong K, Wakayama T, Zhang Y. A role for the elongator complex in zygotic paternal genome demethylation. Nature. 2010;463: 554–558. doi: 10.1038/nature08732 20054296 10.1038/nature08732 PMC2834414

[19] Defraia CT, Wang Y, Yao J, Mou Z. Elongator subunit 3 positively regulates plant immunity through its histone acetyltransferase and radical S-adenosylmethionine domains. BMC Plant Biol. 2013;13: 102 doi: 10.1186/1471-2229-13-102 23856002 10.1186/1471-2229-13-102 PMC3728140

[20] Sidik SM, Huet D, Ganesan SM, Huynh M-H, Wang T, Nasamu AS, et al A Genome-wide CRISPR Screen in Toxoplasma Identifies Essential Apicomplexan Genes. Cell. 2016;166: 1423–1435.e12. doi: 10.1016/j.cell.2016.08.019 27594426 10.1016/j.cell.2016.08.019 PMC5017925

[21] Padgett LR. Investigations into the function of Elp3 in Toxoplasma gondii. [Internet]. Indiana University School of Medicine. 2017 Available: https://scholarworks.iupui.edu/handle/1805/13849

[22] Schmidt EK, Clavarino G, Ceppi M, Pierre P. SUnSET, a nonradioactive method to monitor protein synthesis. Nat Methods. 2009;6: 275–277. doi: 10.1038/nmeth.1314 19305406 10.1038/nmeth.1314

[23] Dadehbeigi N, Dickson AJ. Application of a nonradioactive method of measuring protein synthesis in industrially relevant Chinese hamster ovary cells. Biotechnol Prog. 2013;29: 1043–1049. doi: 10.1002/btpr.1750 23749410 10.1002/btpr.1750

[24] Teske BF, Baird TD, Wek RC. Methods for analyzing eIF2 kinases and translational control in the unfolded protein response. Meth Enzymol. 2011;490: 333–356. doi: 10.1016/B978-0-12-385114-7.00019-2 21266259 10.1016/B978-0-12-385114-7.00019-2

[25] Nakamoto MA, Lovejoy AF, Cygan AM, Boothroyd JC. mRNA pseudouridylation affects RNA metabolism in the parasite Toxoplasma gondii. RNA. 2017; doi: 10.1261/rna.062794.117 28851751 10.1261/rna.062794.117 PMC5689004

[26] Björk GR, Huang B, Persson OP, Byström AS. A conserved modified wobble nucleoside (mcm5s2U) in lysyl-tRNA is required for viability in yeast. RNA. 2007;13: 1245–1255. doi: 10.1261/rna.558707 17592039 10.1261/rna.558707 PMC1924908

[27] Delaunay S, Rapino F, Tharun L, Zhou Z, Heukamp L, Termathe M, et al Elp3 links tRNA modification to IRES-dependent translation of LEF1 to sustain metastasis in breast cancer. J Exp Med. 2016;213: 2503–2523. doi: 10.1084/jem.20160397 27811057 10.1084/jem.20160397 PMC5068235

[28] Frohloff F, Fichtner L, Jablonowski D, Breunig KD, Schaffrath R. Saccharomyces cerevisiae Elongator mutations confer resistance to the Kluyveromyces lactis zymocin. EMBO J. 2001;20: 1993–2003. doi: 10.1093/emboj/20.8.1993 11296232 10.1093/emboj/20.8.1993 PMC125238

[29] Huang B, Lu J, Byström AS. A genome-wide screen identifies genes required for formation of the wobble nucleoside 5-methoxycarbonylmethyl-2-thiouridine in Saccharomyces cerevisiae. RNA. 2008;14: 2183–2194. doi: 10.1261/rna.1184108 18755837 10.1261/rna.1184108 PMC2553728

[30] Lu J, Huang B, Esberg A, Johansson MJO, Byström AS. The Kluyveromyces lactis gamma-toxin targets tRNA anticodons. RNA. 2005;11: 1648–1654. doi: 10.1261/rna.2172105 16244131 10.1261/rna.2172105 PMC1370851

[31] Dewez M, Bauer F, Dieu M, Raes M, Vandenhaute J, Hermand D. The conserved Wobble uridine tRNA thiolase Ctu1-Ctu2 is required to maintain genome integrity. Proc Natl Acad Sci USA. 2008;105: 5459–5464. doi: 10.1073/pnas.0709404105 18391219 10.1073/pnas.0709404105 PMC2291126

[32] Fernández-Vázquez J, Vargas-Pérez I, Sansó M, Buhne K, Carmona M, Paulo E, et al Modification of tRNA(Lys) UUU by elongator is essential for efficient translation of stress mRNAs. PLoS Genet. 2013;9: e1003647 doi: 10.1371/journal.pgen.1003647 23874237 10.1371/journal.pgen.1003647 PMC3715433

[33] Chen C, Huang B, Anderson JT, Byström AS. Unexpected accumulation of ncm(5)U and ncm(5)S(2) (U) in a trm9 mutant suggests an additional step in the synthesis of mcm(5)U and mcm(5)S(2)U. PLoS ONE. 2011;6: e20783 doi: 10.1371/journal.pone.0020783 21687733 10.1371/journal.pone.0020783 PMC3110198

[34] Gajria B, Bahl A, Brestelli J, Dommer J, Fischer S, Gao X, et al ToxoDB: an integrated Toxoplasma gondii database resource. Nucleic Acids Res. 2008;36: D553–556. doi: 10.1093/nar/gkm981 18003657 10.1093/nar/gkm981 PMC2238934

[35] Lowe TM, Chan PP. tRNAscan-SE On-line: integrating search and context for analysis of transfer RNA genes. Nucleic Acids Res. 2016;44: W54–57. doi: 10.1093/nar/gkw413 27174935 10.1093/nar/gkw413 PMC4987944

[36] Lowe TM, Eddy SR. tRNAscan-SE: a program for improved detection of transfer RNA genes in genomic sequence. Nucleic Acids Res. 1997;25: 955–964. 9023104 10.1093/nar/25.5.955 PMC146525

[37] Johansson MJO, Esberg A, Huang B, Björk GR, Byström AS. Eukaryotic wobble uridine modifications promote a functionally redundant decoding system. Mol Cell Biol. 2008;28: 3301–3312. doi: 10.1128/MCB.01542-07 18332122 10.1128/MCB.01542-07 PMC2423140

[38] Grove TL, Lee K-H, St Clair J, Krebs C, Booker SJ. In vitro characterization of AtsB, a radical SAM formylglycine-generating enzyme that contains three [4Fe-4S] clusters. Biochemistry. 2008;47: 7523–7538. doi: 10.1021/bi8004297 18558715 10.1021/bi8004297 PMC2664749

[39] Klassen R, Grunewald P, Thüring KL, Eichler C, Helm M, Schaffrath R. Loss of anticodon wobble uridine modifications affects tRNA(Lys) function and protein levels in Saccharomyces cerevisiae. PLoS ONE. 2015;10: e0119261 doi: 10.1371/journal.pone.0119261 25747122 10.1371/journal.pone.0119261 PMC4352028

[40] Ranjan N, Rodnina MV. tRNA wobble modifications and protein homeostasis. Translation (Austin). 2016;4: e1143076 doi: 10.1080/21690731.2016.1143076 27335723 10.1080/21690731.2016.1143076 PMC4909423

[41] Nedialkova DD, Leidel SA. Optimization of Codon Translation Rates via tRNA Modifications Maintains Proteome Integrity. Cell. 2015;161: 1606–1618. doi: 10.1016/j.cell.2015.05.022 26052047 10.1016/j.cell.2015.05.022 PMC4503807

[42] Zinshteyn B, Gilbert WV. Loss of a conserved tRNA anticodon modification perturbs cellular signaling. PLoS Genet. 2013;9: e1003675 doi: 10.1371/journal.pgen.1003675 23935536 10.1371/journal.pgen.1003675 PMC3731203

[43] Walden WE, Godefroy-Colburn T, Thach RE. The role of mRNA competition in regulating translation. I. Demonstration of competition in vivo. J Biol Chem. 1981;256: 11739–11746. 7298628

[44] Lodish HF, Jacobsen M. Regulation of hemoglobin synthesis. Equal rates of translation and termination of—and -globin chains. J Biol Chem. 1972;247: 3622–3629. 4624122

[45] Tyagi K, Pedrioli PGA. Protein degradation and dynamic tRNA thiolation fine-tune translation at elevated temperatures. Nucleic Acids Res. 2015;43: 4701–4712. doi: 10.1093/nar/gkv322 25870413 10.1093/nar/gkv322 PMC4482078

[46] Schaffrath R, Leidel SA. Wobble uridine modifications-a reason to live, a reason to die?! RNA Biol. 2017; 1–14. doi: 10.1080/15476286.2017.1295204 28277930 10.1080/15476286.2017.1295204 PMC5699542

[47] Esseiva AC, Naguleswaran A, Hemphill A, Schneider A. Mitochondrial tRNA import in Toxoplasma gondii. J Biol Chem. 2004;279: 42363–42368. doi: 10.1074/jbc.M404519200 15280394 10.1074/jbc.M404519200

[48] Kaneko T, Suzuki T, Kapushoc ST, Rubio MA, Ghazvini J, Watanabe K, et al Wobble modification differences and subcellular localization of tRNAs in Leishmania tarentolae: implication for tRNA sorting mechanism. EMBO J. 2003;22: 657–667. doi: 10.1093/emboj/cdg066 12554666 10.1093/emboj/cdg066 PMC140750

[49] Paris Z, Rubio MAT, Lukes J, Alfonzo JD. Mitochondrial tRNA import in Trypanosoma brucei is independent of thiolation and the Rieske protein. RNA. 2009;15: 1398–1406. doi: 10.1261/rna.1589109 19465685 10.1261/rna.1589109 PMC2704085

[50] Donald RG, Roos DS. Stable molecular transformation of Toxoplasma gondii: a selectable dihydrofolate reductase-thymidylate synthase marker based on drug-resistance mutations in malaria. Proc Natl Acad Sci USA. 1993;90: 11703–11707. 8265612 10.1073/pnas.90.24.11703 PMC48052

[51] Bhatti MM, Sullivan WJ. Histone acetylase GCN5 enters the nucleus via importin-alpha in protozoan parasite Toxoplasma gondii. J Biol Chem. 2005;280: 5902–5908. doi: 10.1074/jbc.M410656200 15591057 10.1074/jbc.M410656200

[52] Donald RG, Roos DS. Gene knock-outs and allelic replacements in Toxoplasma gondii: HXGPRT as a selectable marker for hit-and-run mutagenesis. Mol Biochem Parasitol. 1998;91: 295–305. 9566522 10.1016/s0166-6851(97)00210-7

[53] Ufermann C-M, Müller F, Frohnecke N, Laue M, Seeber F. Toxoplasma gondii plaque assays revisited: Improvements for ultrastructural and quantitative evaluation of lytic parasite growth. Exp Parasitol. 2016; doi: 10.1016/j.exppara.2016.12.015 28011168 10.1016/j.exppara.2016.12.015

[54] Chaparas SD, Schlesinger RW. Plaque assay of Toxoplasma on monolayers of chick embryo fibroblasts. Proc Soc Exp Biol Med. 1959;102: 431–437. 13809203 10.3181/00379727-102-25275

[55] Roos DS, Donald RG, Morrissette NS, Moulton AL. Molecular tools for genetic dissection of the protozoan parasite Toxoplasma gondii. Methods Cell Biol. 1994;45: 27–63. 7707991 10.1016/s0091-679x(08)61845-2

[56] Dvorak JA, Howe CL. Toxoplasma gondii-vertebrate cell interactions. II. The intracellular reproductive phase. J Protozool. 1979;26: 114–117. 480273 10.1111/j.1550-7408.1979.tb02742.x

[57] Ramirez M, Wek RC, Hinnebusch AG. Ribosome association of GCN2 protein kinase, a translational activator of the GCN4 gene of Saccharomyces cerevisiae. Mol Cell Biol. 1991;11: 3027–3036. 2038314 10.1128/mcb.11.6.3027-3036.1991 PMC360137

[58] Narasimhan J, Joyce BR, Naguleswaran A, Smith AT, Livingston MR, Dixon SE, et al Translation regulation by eukaryotic initiation factor-2 kinases in the development of latent cysts in Toxoplasma gondii. J Biol Chem. 2008;283: 16591–16601. doi: 10.1074/jbc.M800681200 18420584 10.1074/jbc.M800681200 PMC2423249

[59] Schneider CA, Rasband WS, Eliceiri KW. NIH Image to ImageJ: 25 years of image analysis. Nat Methods. 2012;9: 671–675. 22930834 10.1038/nmeth.2089 PMC5554542

