# Supplementary figures and images for: Elp3 and RlmN: A tale of two mitochondrial tail-anchored radical SAM enzymes in Toxoplasma gondii
Source: PLoS One. 2018 Jan 2;13(1):e0189688. doi: 10.1371/journal.pone.0189688 (PMC5749711; doi:10.1371/journal.pone.0189688)

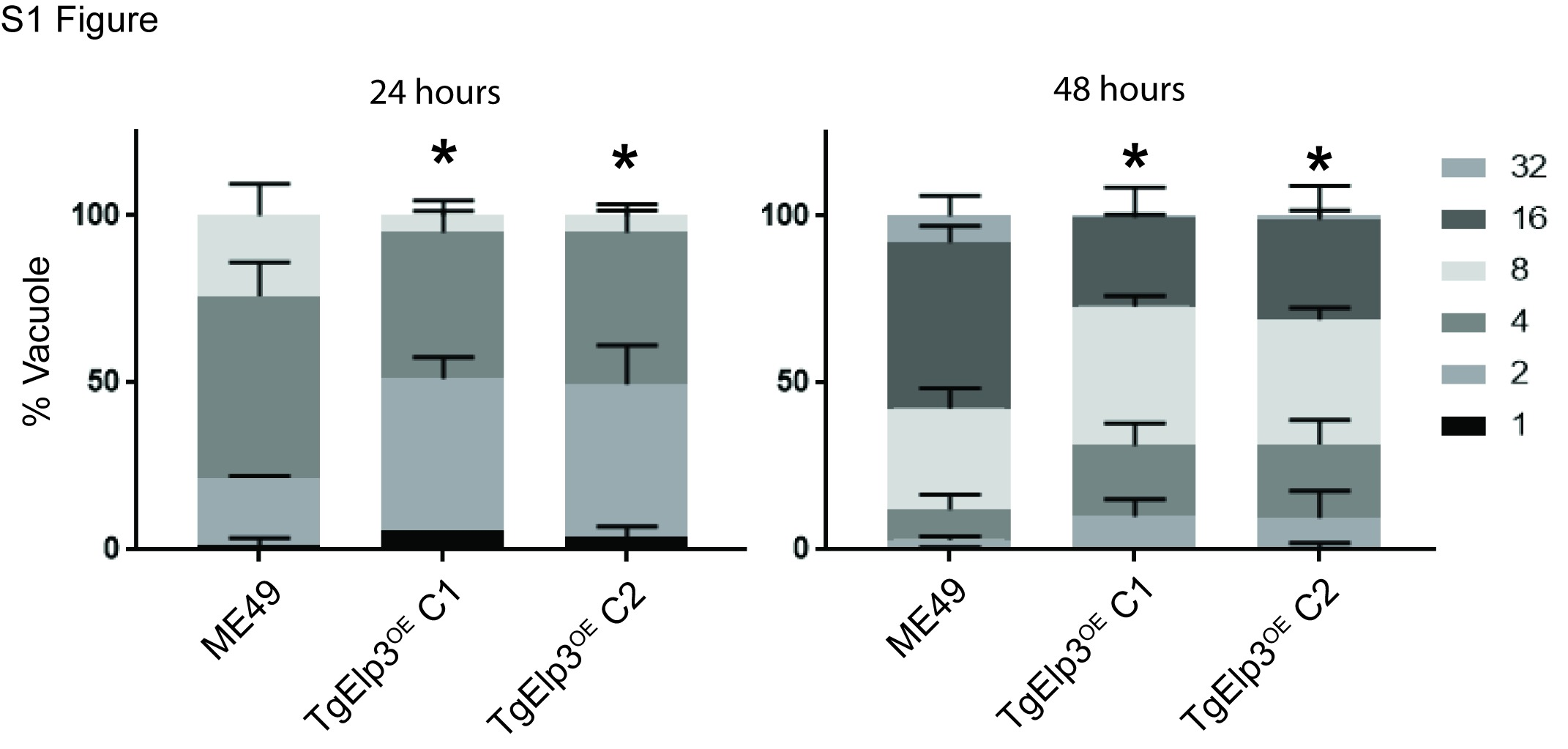

Supplement: S1 Fig — Replication rate was assessed in the parental ME49 and two independent TgElp3 overexpressing ME49 parasite strains (TgElp3OE C1 and C2). Doubling assays were performed at 24 and 48 hours; the number of parasites in 100 random vacuoles was quantified and the percentage of vacuoles containing the designated number of parasites ± s.d. is shown, *P<0.05 (two-way ANOVA). (TIF) [file pone.0189688.s001.tif]

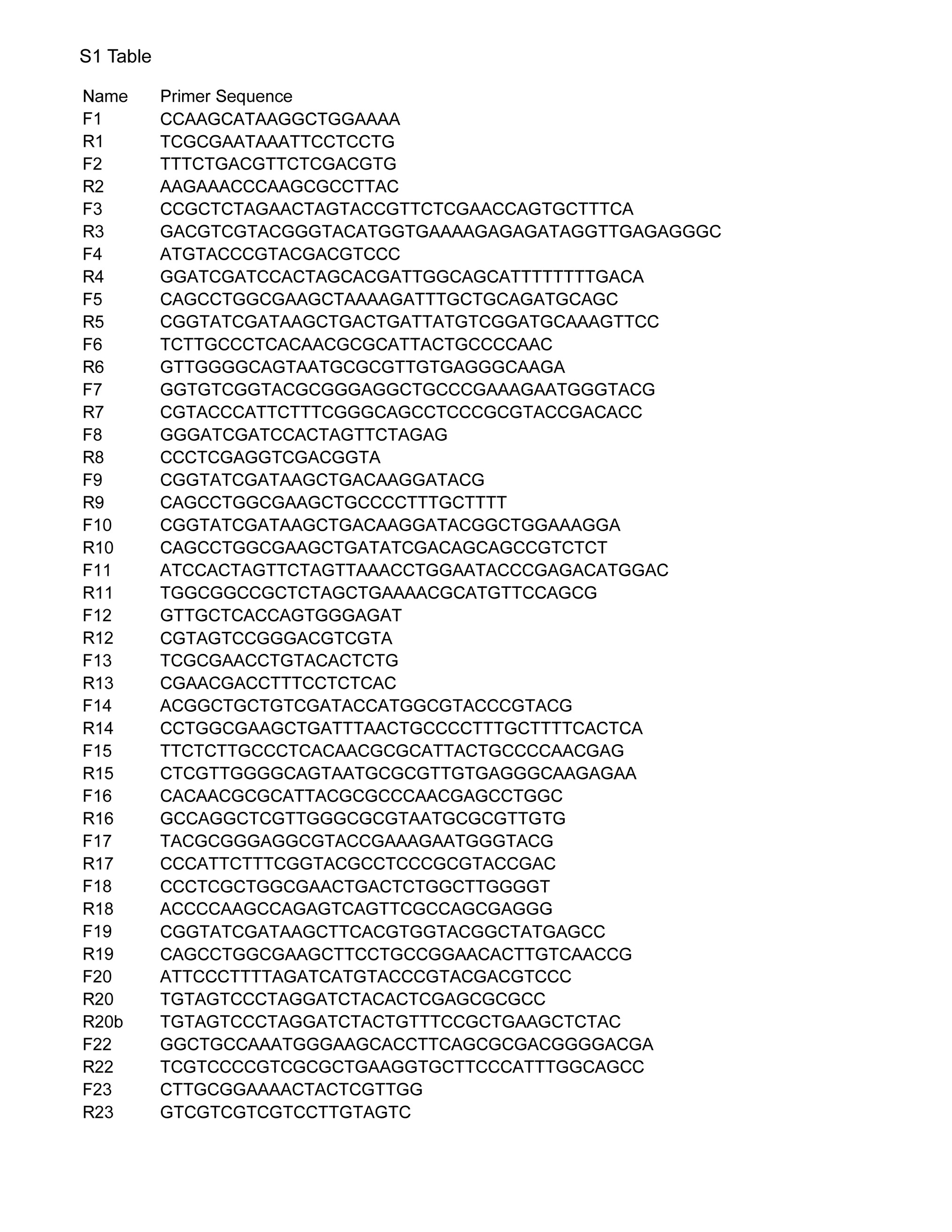

Supplement: S1 Table — (TIF) [file pone.0189688.s002.tif]
